# IncRNA AC004943.2 regulates miR‐135a‐5p and PTK2/P13K axis to promote laryngeal squamous cell carcinoma progression

**DOI:** 10.1002/ccs3.12016

**Published:** 2024-02-09

**Authors:** Xiaowen Zhu, Wenming Dong, Meijia Zhang

**Affiliations:** ^1^ Department of General Surgery Fourth Ward The First Affiliated Hospital of Jiamusi University Jiamusi Heilongjiang Province China; ^2^ Department of Anesthesiology Shenzhen Baoan Hospital of TCM Shenzhen Guangdong Province China; ^3^ Department of Otolaryngology The First Affiliated Hospital of Jiamusi University Jiamusi Heilongjiang Province China

**Keywords:** AC004943.2, laryngeal squamous cell carcinoma, miR‐135a‐5p, PI3K, PTK2

## Abstract

Long noncoding RNAs (lncRNAs) are involved in regulatory processes in laryngeal squamous cell carcinoma (LSCC) at posttranscriptional epigenetic modification level. Yet, the function and underlying mechanism behind lncRNA AC004943.2 in LSCC is still obscure. Therefore, the potential role of AC004943.2 in LSCC progression was investigated. The expression of gene or protein was tested by real‐time quantitative polymerase chain reaction and western blot. MTT, colony formation, wound healing, and transwell experiments were applied to detect LSCC cell viability, proliferation, migration and invasion, respectively. The interaction among AC004943.2, miR‐135a‐5p, and protein tyrosine kinase 2 (PTK2) were analyzed by bioinformatics prediction and luciferase assay. AC004943.2 was highly expressed in LSCC cells compared with normal human bronchial epithelial cells, while miR‐135a‐5p was lowly expressed. AC004943.2 knockdown or miR‐135a‐5p overexpression inhibited LSCC cell viability, proliferation, migration and invasion. Mechanistically, AC004943.2 increased PTK2 expression in LSCC cells by sponging miR‐135a‐5p. Furthermore, miR‐135a‐5p knockdown inverted the inhibitory effect of AC004943.2 silencing on LSCC cell malignant behaviors. MiR‐135a‐5p upregulation attenuated the PTK2/PI3K pathway to inhibit progression of LSCC. AC004943.2 facilitated the cancerous phenotypes of LSCC cells by activating the PTK2/PI3K pathway through targeting miR‐135a‐5p, which furnished a therapeutic candidate for LSCC treatment.

## Graphical abstract

AC004943.2 facilitated the proliferation and migration and invasion of laryngeal squamous cell carcinoma cells through sponging miR‐135a‐5p and then activating the PTK2/PI3K pathway.

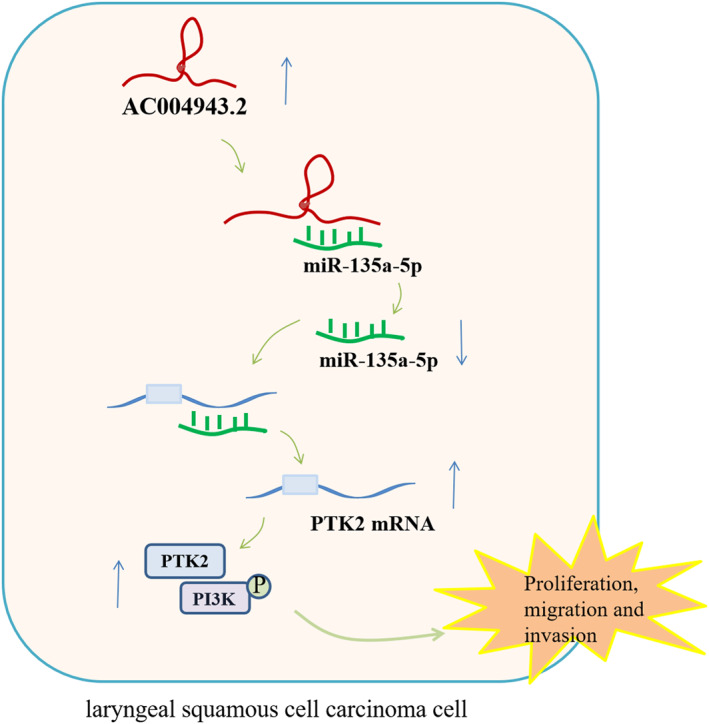

## 1 INTRODUCTION

Laryngeal squamous cell carcinoma (LSCC) is a prevalent kind of head and neck cancer.^1^ The frequency and the mortality rate of LSCC have been rising in recent years. Although the diagnostic technique has improved, over 60% of LSCC patients are still detected in the late stages, and clinical survival is dismal.^2^, ^3^ Therefore, it is imperative to search new biomarkers to diagnose and investigate the underlying mechanism of LSCC progression.

Long noncoding RNAs (lncRNAs) refer to noncoding RNAs with a length over to 200 nucleotides, which have been discovered and occupy a significantly large proportion of the human genome.^4^ LncRNAs can play a crucial role in the majority of key cellular processes involved in the maintenance of cellular homeostasis by regulating various molecular mechanisms.^5^ Recent studies have proved that the deregulation of lncRNAs has been associated with various chronic diseases and human malignancies, including LSCC.^5^, ^6^, ^7^ Kong et al. pointed out that lncRNA AC016773.1 and C00299 participated in LSCC progression.^8^ Cao et al. identified that lncRNA IGKJ2‐MALLP2 inhibited LSCC progression by sponging miR‐1911‐3p.^6^ Interestingly, it has been reported that lncRNA AC004943.2 was upregulated in LSCC.^9^ Nevertheless, the exact role and the mechanism of lncRNA AC004943.2 in LSCC is still unknown.

MicroRNAs (miRNAs) were a type of endogenous noncoding small RNAs with around 22 nucleotides in length, which are ubiquitously expressed and participate in modulating gene expression. Recent studies showed that miRNAs dysregulation play critical roles in carcinogenesis. As widely described, miRNAs are involved in LSCC progression.^10^ For instance, miR‐29a upregulation inhibited LSCC progression by targeting STAT3.^11^ In addition, miR‐125b could suppress LSCC cell growth and migration by targeting STAT3.^12^ Of note, it has been widely described that miR‐135a‐5p can function as an orchestrator of various cellular processes, including the differentiation, regeneration, and tumorigenesis.^13^, ^14^ MiR‐135a‐5p downregulation was observed in several cancers, and its low expression was linked to poor overall prognosis in gastric cancer. MiR‐135a‐5p targets various genes of in tumors, including FOXO1, KIFC1, and ERRalpha, which are involved in cell invasion and sensitivity to chemotherapy.^15^, ^16^, ^17^ Herein, by using the bioinformatic prediction, we noticed that AC004943.2 had potential binding sites to miR‐135a‐5p, and miR‐135a‐5p had potential binding sites to protein tyrosine kinase 2 (PTK2). The function and the underlying mechanism of miR‐135a have not been explored yet in LSCC.

In this study, we performed a detailed investigation of AC004943.2 in regulating the miR‐135a‐5p/PTK2 axis to promote LSCC progression. Our results extend the understanding of AC004943.2 in the progression of LSCC, which provides effective targets for LSCC diagnoses and therapy.

## 2 MATERIALS AND METHODS

### 2.1 Cell culture

Normal human bronchial epithelial cells 16HBE and LSCC cells TU686, AMC‐HN‐8, LSC‐1, TU177, and M4E were acquired from iCell Bioscience Inc (Shanghai, China). All cells were cultured in DMEM (Gibco, USA) containing 10% FBS (Carlsbad, USA).

### 2.2 Cell transfection

Cells were seeded in 6‐well plates at a density of 1 × 10^5^ cells/well and cultured for 24 h. Cells were then transfected with sh‐AC004943.2, miR‐135a‐5p inhibitor, miR‐135a‐5p mimics, OE‐PTK2, and their corresponding negative controls using Lipofectamine 2000 (Invitrogen, USA). The plasmids were synthesized and purchased from GenePharma (Shanghai, China). After 48 h, cells were gathered for follow‐up analysis.

### 2.3 Real‐time quantitative polymerase chain reaction

The total RNA was extracted using TRIzol reagent (Invitrogen). For mRNA, cDNA was generated by the High‐Capacity RNA‐to‐cDNA Kit (Applied Biosystems, USA). The SYBR qPCR master mix (TOYOBO, Japan) was used to measure the fluorescence intensity of PCR reactions. To detect the expression of miRNA, quantitative PCR were measured by TaqMan Universal Master Mix II (Applied Biosystems). GAPDH and U6 were applied as internal controls for mRNA or miRNA, respectively. The primers were listed as follows:

PTK2: F, 5′‐CAGGGTCCGATTGGAAACCA‐3′, R, 5′‐ CTGAAGCTTGACACCCTCGT‐3′; GAPDH: F, 5′‐CTGGGCTACACTGAGCACC‐3′, R, 5′‐AAGTGGTCGTTGAGGGCAATG‐3′; miR‐135a‐5p: F, 5′‐TTGAAGAAACCCTTGAGGAA‐3′, R, 5′‐CTGCCGAATAATCTCCATCT‐3′; AC004943.2: F, 5′‐CACTTCTGCAGGAACACCGA ‐3′, R, 5′‐GTGGAGACTGAATGGCCCTC‐3′; and U6: F, 5′‐AATTTGAAGAAGCGGTTGC‐3′, R, 5′‐GTGGAACTGGGAGAACAAG‐3′.

### 2.4 3‐(4,5‐Dimethylthiazol‐2‐yl)‐2,5‐diphenyltetrazolium bromide (MTT) assay

Cell viability was detected using MTT assay. Cells were seeded in 96‐well plates at a density of 1 × 10^3^/well and transfected with indicated plasmids for 48 h. Cells were then incubated with 20 μL MTT (Sigma‐Aldrich, Shanghai, China) for 4 h, followed by the precipitate dissolving in 100 μL DMSO (Sigma‐Aldrich). The sample was quantified at 490 nm absorbance using a microplate spectrophotometer (Bio‐Tek, USA).

### 2.5 Wound healing assay

Cells were inoculated and grown to reach 90% confluence, and then scratched in the single layer with the tip of pipette. Cells were subsequently put in an incubator for additional 4 h, and the images were captured. Digital photographs were obtained from a Carl Zeiss light microscope (Axio Observer A1, Germany), and the scratch area was measured by Image‐J software.

### 2.6 Transwell assay

Cells were transfected with indicated plasmids and planted in the upper compartment containing Matrigel‐coated membrane (Cat. No. #354230, Thermo Fisher Scientific) and incubated in DMEM without serum. The lower compartment was incorporated with FBS‐containing medium. Invading cells in the down chamber were fixed with methanol after cultivation. The invaded cells were dyed with crystal violet and captured using a Carl Zeiss light microscope (Axio Observer A1).

### 2.7 Colony formation assay

Cells were inoculated for colony forming assay. After 14 days of cell growth, the cell clones were formed and examined. The clones were then immobilized with menthol and dyed with 0.1% crystal violet at ambient temperature. Then, all colonies were preserved with ethanol, and they were dyed with crystal violet for 2 h. Finally, they were captured under a Carl Zeiss light microscope (Axio Observer A1).

### 2.8 Western blot

Protein samples were extracted by using ice‐cold lysis buffer (Beyotime, China) containing protease inhibitors (Cell Signaling Technology, Boston, USA). The protein concentrations were determined using a BCA assay. Protein samples (20 μg) were fractionated on 10% SDS‐PAGE and wet‐transferred onto PVDF membranes. The membranes were incubated with specific antibodies against anti‐PTK2 (ab271836), PI3K (ab302959), p‐PI3K (ab278545), and GAPDH (ab9485) overnight. All these antibodies were purchased from Abcam (Cambridge, UK). The membranes were then incubated with HRP‐labeled corresponding secondary antibodies at room temperature for 1 h. Finally, protein expression was performed using a chemiluminescent instrument (Bio‐Rad, USA).

### 2.9 Dual luciferase reporter assay

The Starbase online database was used to predict specific binding sites of miR‐135a‐5p. The wild type (WT) and the mutant (MUT) 3′‐untranslated region (UTR) of PTK2 and AC004943.2 were cloned in Pmir‐GLO dual luciferase miRNA target expression vectors (Promega, USA). Cells were co‐transfected with the WT/MUT plasmid of PTK2, AC004943.2, and miR‐135a‐5p or miR‐135a‐5p‐NC. Cells were collected, and the luciferase activity was assessed using the dual‐luciferase detection kit (E1910, Promega).

### 2.10 Statistical analysis

GraphPad Prism 7 (GraphPad, USA) was used to examine the statistical data. Data were presented as mean ± standard deviation (SD) from at least 3 independent experiments. Student's *t‐test* was used to examine the differences between the two groups, while one‐way ANOVA followed by Tukey's post hoc test was applied to analyze the differences across multiple groups. The *p* values less than 0.05 were regarded as significant.

## 3 RESULTS

### 3.1 AC004943.2 promoted LSCC progression

**FIGURE 1:**
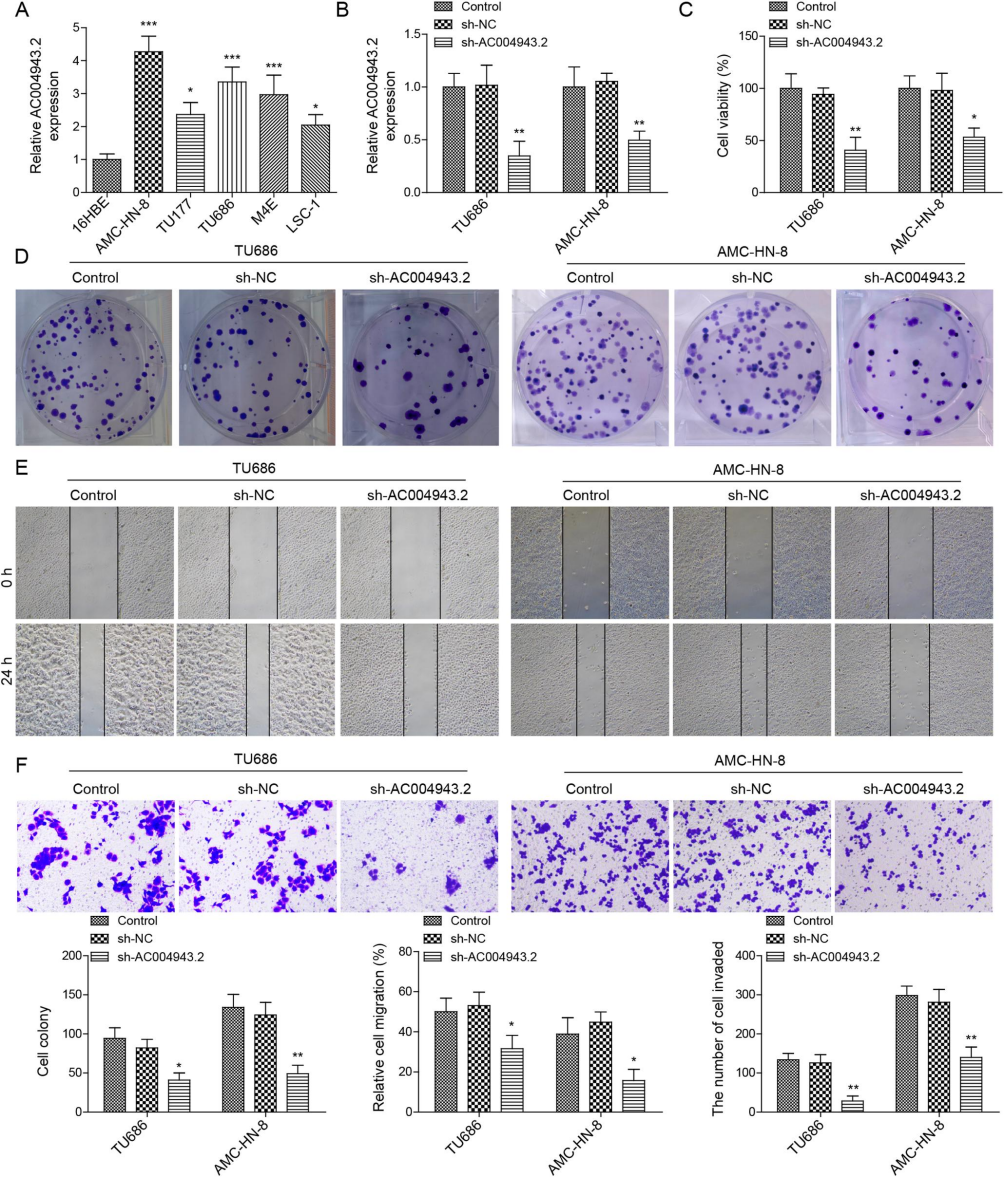
Knockdown of AC004943.2 inhibited LSCC progression. (A) RT‐qPCR was utilized to measure AC004943.2 in 16HBE and different LSCC cells. (B) Knockdown efficiency of AC004943.2 were examined through RT‐qPCR. (C) MTT was utilized to assess LSCC cell viability after AC004943.2 knockdown. (D) Colony formation was employed to evaluate the effects of AC004943.2 knockdown on LSCC proliferation. (E) Wound healing was utilized to analyze the effects of AC004943.2 knockdown on LSCC migration. (F) Transwell assay was applied to investigate LSCC invasion ability after inhibiting AC004943.2. *p < 0.05, **p < 0.01, and ***p < 0.001.

To examine the potential function of AC004943.2 in LSCC, we detected AC004943.2 mRNA level in various LSCC cell lines. As demonstrated in Figure 1A, AC004943.2 was highly expressed in LSCC cells compared with normal human bronchial epithelial cells, particularly in TU686 and AMC‐HN‐8 cells, which were employed for subsequent experiments. Then, we induced AC004943.2 knockdown in TU686 and AMC‐HN‐8 cells (Figure 1B). As shown in Figure 1C,D, knockdown of AC004943.2 suppressed LSCC cell viability and proliferation. Wound healing assay results subsequently displayed that LSCC cell migration was repressed by AC004943.2 silencing (Figure 1E). Moreover, AC004943.2 downregulation remarkedly reduced LSCC cell invasion (Figure 1F). Altogether, our results indicated that endogenous AC004943.2 might promote LSCC progression.

### 3.2 AC004943.2 targeted miR‐135a‐5p in LSCC

**FIGURE 2:**
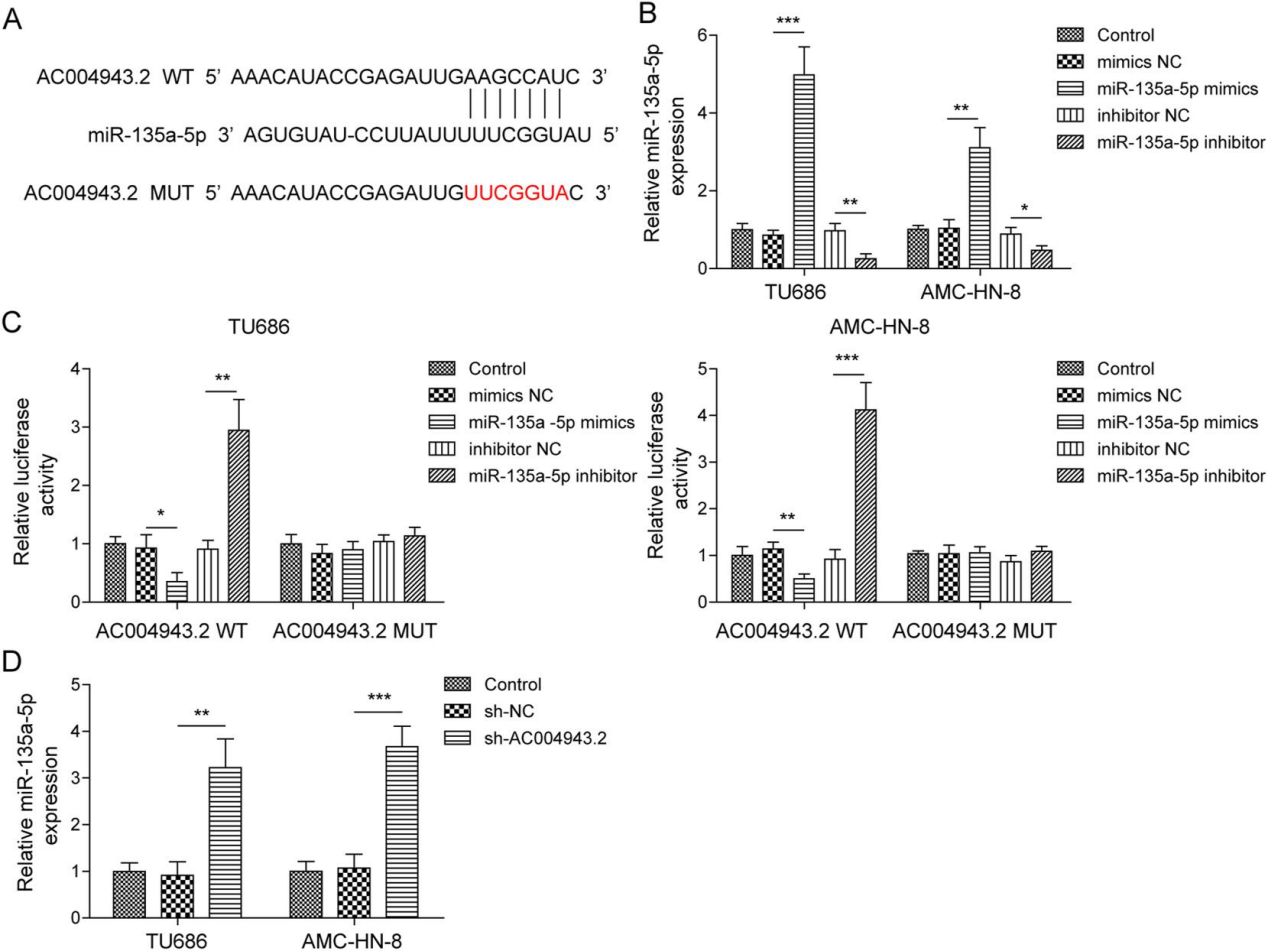
AC004943.2 targeted miR‐135a‐5p in LSCC. (A) The Starbase database was applied to identify the interaction between miR‐135a‐5p and AC004943.2. (B) RT‐qPCR was utilized to test miR‐135a‐5p level. (C) The interaction between AC004943.2 and miR‐135a‐5p was assessed by dual luciferase reporter assay. (D) Effect of AC004943.2 knockdown on miR‐135a‐5p level was measured by RT‐qPCR. *p < 0.05, **p < 0.01, and ***p < 0.001.

Then, we investigated the mechanism of AC004943.2 in LSCC. The Starbase database was applied to screen potential targets of AC004943.2. Bioinformatics results predicated that miR‐135a‐5p was a prospective target gene of AC004943.2 (Figure 2A). Then, we used miR‐135a‐5p mimics or its inhibitor, which could increase or reduce miR‐135a‐5p expression, respectively (Figure 2B). As confirmed by dual luciferase reporter assay, miR‐135a‐5p overexpression/inhibition significantly reduced/increased the luciferase activity of the AC004943.2 WT group but had no significance on the luciferase activity of AC004943.2 MUT (Figure 2C), indicating that AC004943.2 had a mutual binding site for miR‐135a‐5p. It was subsequently revealed that knockdown of AC004943.2 could enhance miR‐135a‐5p level (Figure 2D). Taken together, AC004943.2 targeted miR‐135a‐5p and reduced its level in LSCC.

### 3.3 miR‐135a‐5p suppressed LSCC progression

**FIGURE 3:**
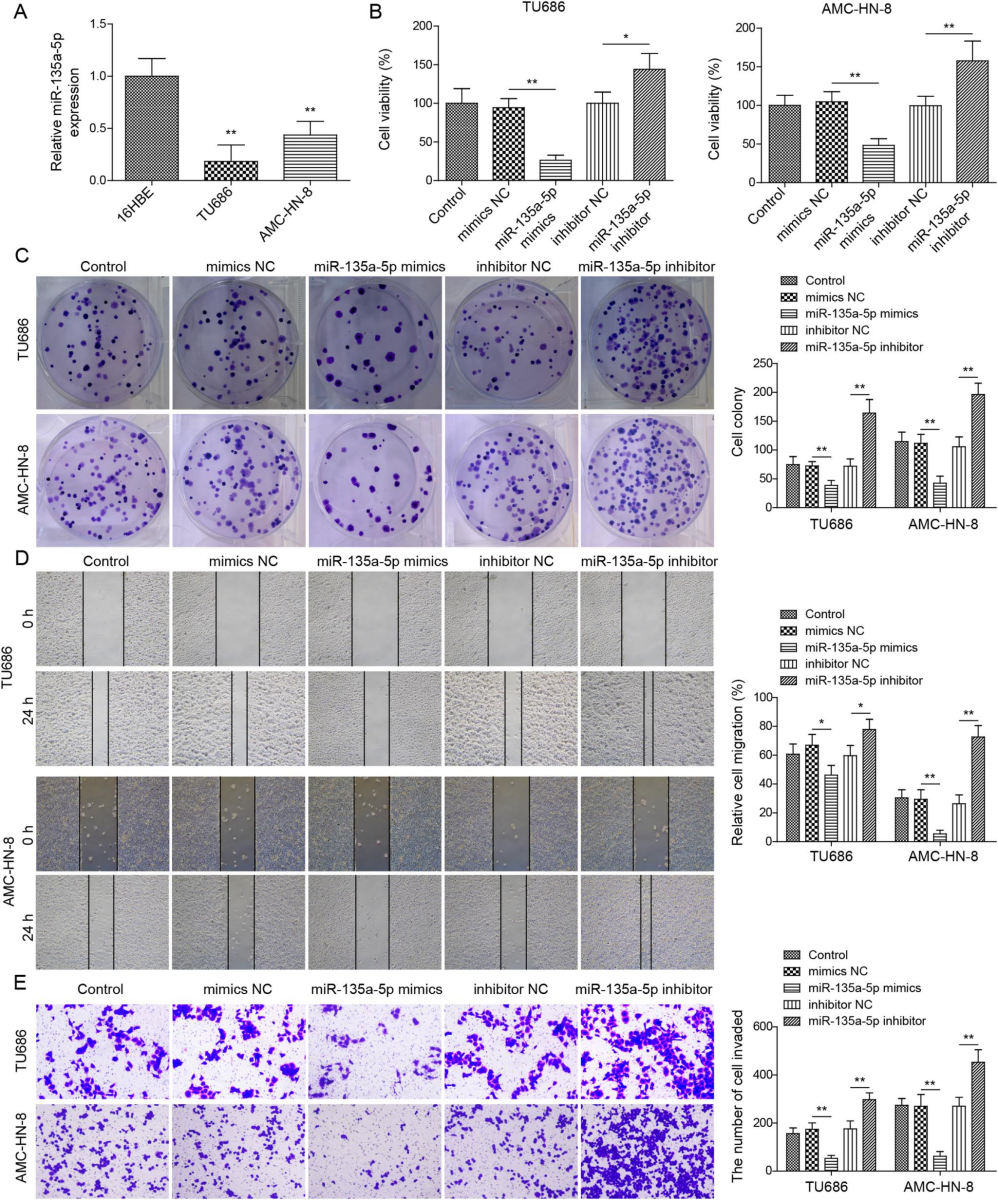
MiR‐135a‐5p suppressed LSCC progression. (A) RT‐qPCR was utilized to examine miR‐135a‐5p level. (B) MTT was utilized to evaluate cell viability. (C) Colony formation was utilized to evaluate cell proliferation. (D) Wound healing was adopted for the cell migration ability test. (E) Transwell was utilized to investigate cell invasion. *p < 0.05, **p < 0.01, and ***p < 0.001.

As shown in Figure 3A, miR‐135a‐5p was lowly expressed in TU686 and AMC‐HN‐8 cell lines compared to 6HBE cells. To determine the function of miR‐135a‐5p in regulating LSCC progression, miR‐135a‐5p overexpression/knockdown was induced in TU686 and AMC‐HN‐8 cells. Functional experiments subsequently showed that miR‐135a‐5p mimics transfection markedly reduced LSCC cell viability and proliferation, while miR‐135a‐5p inhibitor transfection presented the opposite effects (Figure 3B,C). In addition, miR‐135a‐5p upregulation inhibited LSCC cell migration, whereas miR‐135a‐5p inhibition promoted cell migration (Figure 3D). Furthermore, miR‐135a‐5p overexpression suppressed LSCC cell invasion, whereas miR‐135a‐5p silencing had opposite effects (Figure 3E). Collectively, our results implied that miR‐135a‐5p suppressed LSCC progression.

### 3.4 AC004943.2 facilitated LSCC progression through inhibition of miR‐135a‐5p

**FIGURE 4:**
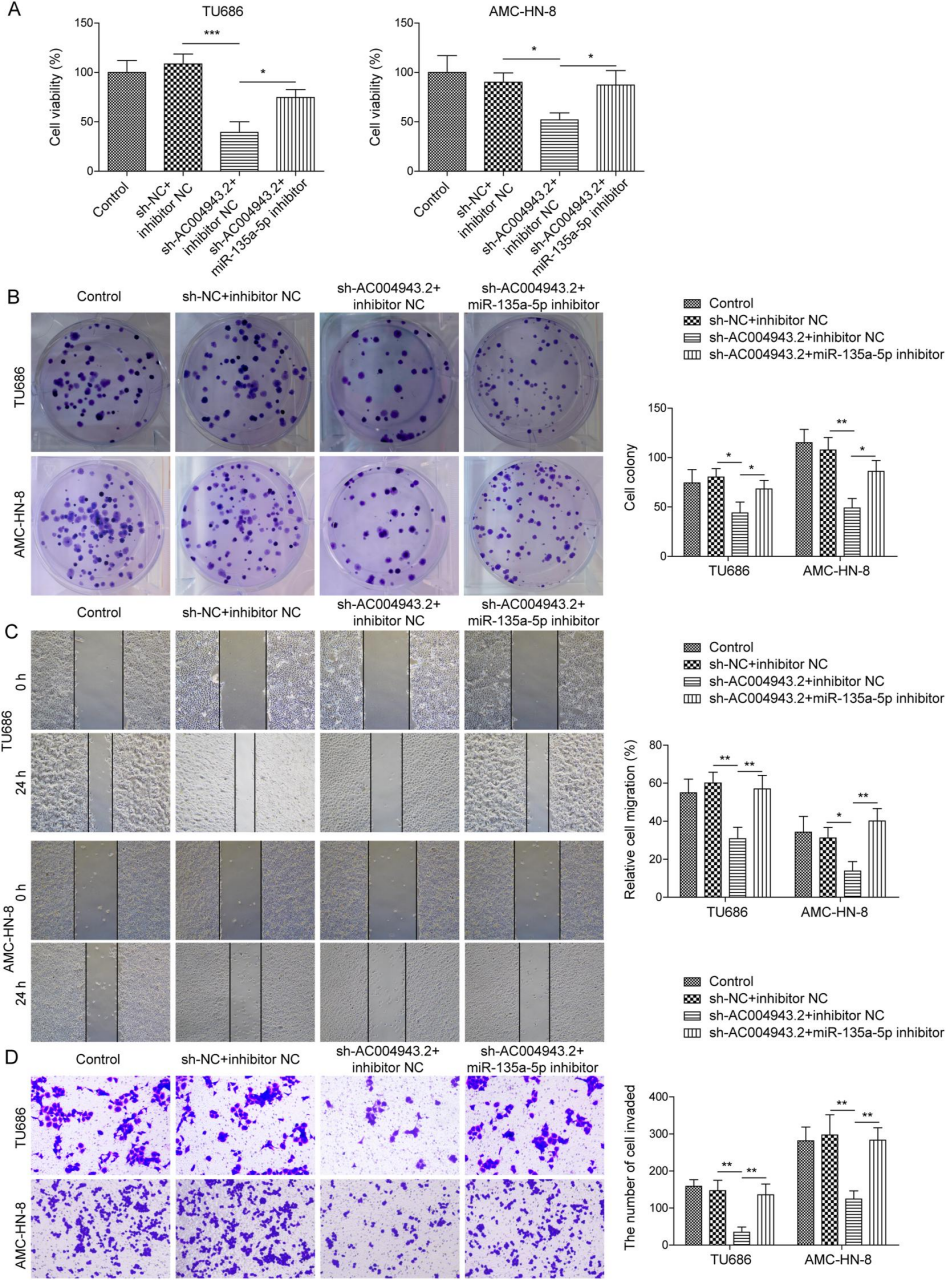
AC004943.2 facilitated LSCC progression through miR‐135a‐5p inhibition. (A) MTT was utilized to evaluate cell viability. (B) Colony formation was conducted for testing cell proliferation. (C) Wound healing was utilized to analyze cell migration. (D) Transwell assay was adopted for cell invasion ability test. *p < 0.05, **p < 0.01, and ***p < 0.001.

To explore whether AC004943.2 promotes LSCC progression through miR‐135a‐5p, both AC004943.2 knockdown and miR‐135a‐5p knockdown were induced in TU686 and AMC‐HN‐8 cells, respectively. It turned out that knockdown of AC004943.2 inhibited LSCC cell viability and proliferation, whereas this inhibitory effect was reversed upon the miR‐135a‐5p inhibitor (Figure 4A,B). Additionally, we noticed that AC004943.2 knockdown attenuated cell migration of LSCC cells, whereas this outcome was counteracted upon the miR‐135a‐5p inhibitor (Figure 4C). Moreover, we found that knockdown of AC004943.2 reduced cell invasion of LSCC cells, whereas the miR‐135a‐5p inhibitor reversed this effect (Figure 4D). Taken together, these data indicated that LSCC progression repressed by AC004943.2 knockdown were abolished by miR‐135a‐5p silencing.

### 3.5 MiR‐135a‐5p targeted PTK2

**FIGURE 5:**
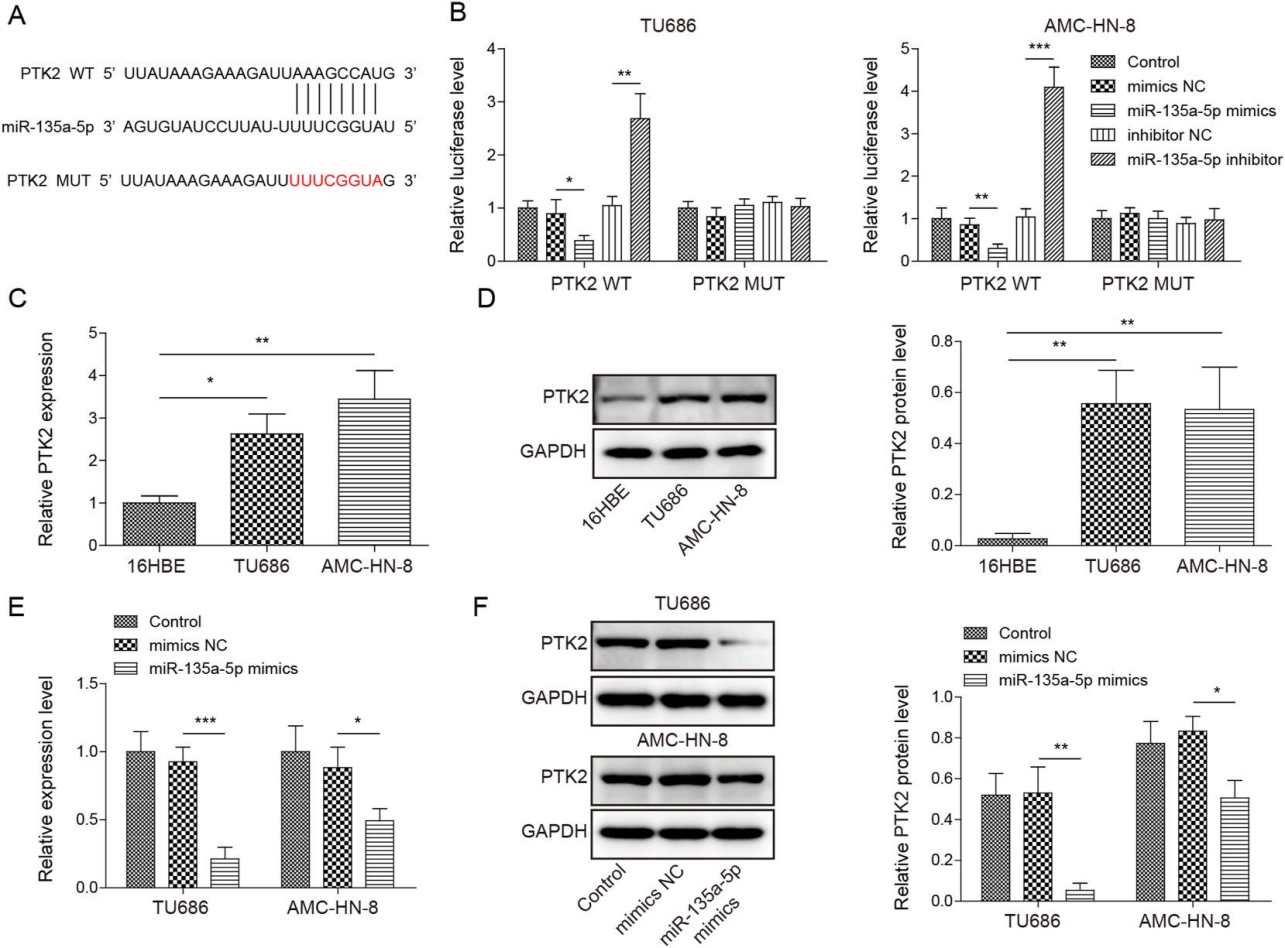
MiR‐135a‐5p targeted PTK2 in LSCC. (A) The miR‐135a‐5p binding site on PTK2 was analyzed by the Starbase database. (B) The interaction between PTK2 and miR‐135a‐5p was assessed through dual luciferase reporter assay. (C) RT‐qPCR was utilized to evaluate PTK2 mRNA level. (D) Western blot was applied to analyze PTK2 protein level. (E) RT‐qPCR was utilized to assess PTK2 mRNA level. (F) Western blot was utilized to assess miR‐135a‐5p mimics on PTK2 protein level. *p < 0.05, **p < 0.01, and ***p < 0.001.

To explore the mechanism of miR‐135a‐5p in LSCC, we used the Starbase database to search for miR‐135a‐5p potential targets. As revealed in Figure 5A, miR‐135a‐5p had potential binding sites to PTK2. Dual luciferase reporter assay results indicated that miR‐135a‐5p mimics/inhibitor transfection remarkedly inhibited/promoted the luciferase activity of the PTK2 WT group, whereas neither miR‐135a‐5p mimics and not its inhibitor had any effect on the luciferase activity of the PTK2 MUT group (Figure 5B), suggesting that PTK2 directly bounded with miR‐135a‐5p. Furthermore, we detected PTK2 in LSCC cells. As depicted in Figure 5C,D, PTK2 was highly expressed in TU686 and AMC‐HN‐8 cells compared to 16HBE cells. It was also turned out that miR‐135a‐5p overexpression significantly reduced PTK2 mRNA and protein levels in LSCC cells (Figure 5E,F). To sum up, these data implied that miR‐135a‐5p downregulated PTK2 expression in LSCC cells.

### 3.6 MiR‐135a‐5p inhibited LSCC progression by inactivating the PTK2/PI3K pathway

**FIGURE 6:**
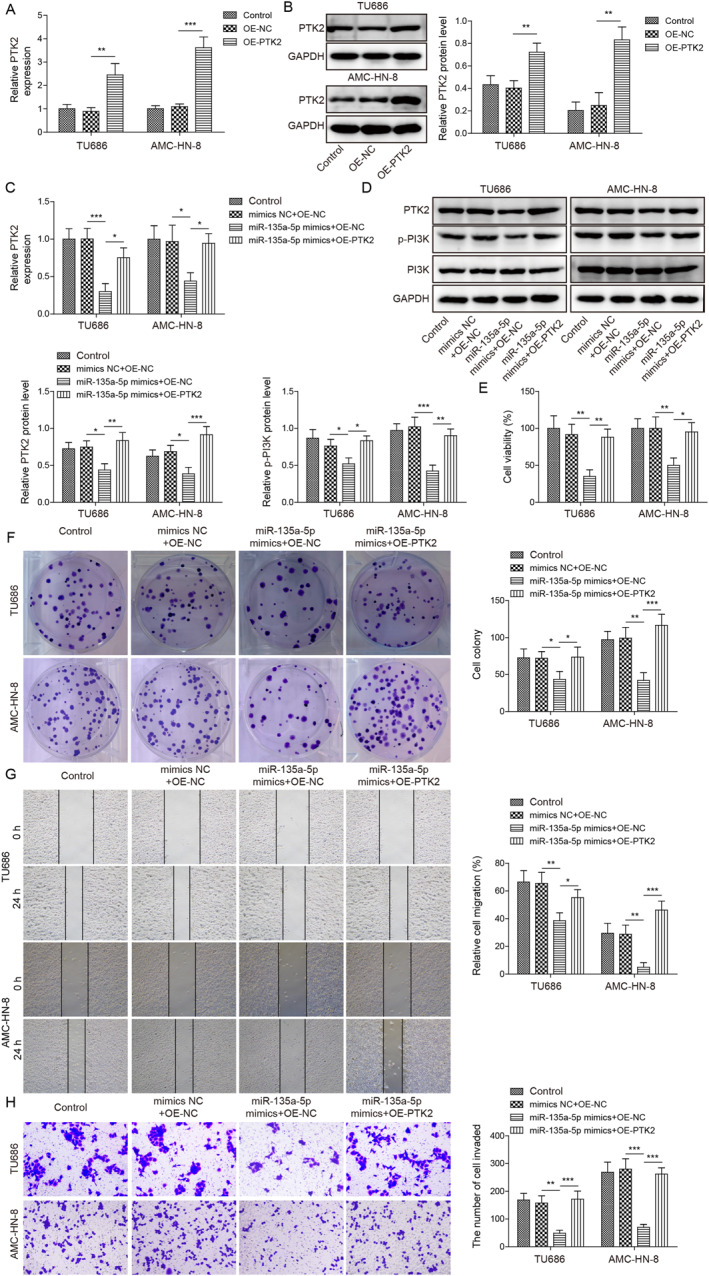
MiR‐135a‐5p attenuated the PTK2/PI3K pathway to inhibition of LSCC. (A) RT‐qPCR examined PTK2 mRNA level. (B) PTK2 expression was detected through western blot. (C) RT‐qPCR assessed the PTK2 mRNA level. (D) Western blot examined PTK2, PI3K, and p‐PI3K protein levels. (E) MTT was utilized to evaluate cell viability. (F) Colony formation was utilized to evaluate cell proliferation. (G) Wound healing was utilized to analyze cell migration. (H) Transwell was utilized to investigate cell invasion. *p < 0.05, **p < 0.01, and ***p < 0.001.

To investigate whether miR‐135a‐5p regulates LSCC development by targeting PTK2, we evaluated PTK2 expression upon co‐transfecting miR‐135a‐5p mimics and the PTK2 overexpression plasmid in LSCC cells. As illustrated in Figure 6A,B, OE‐PTK2 transfection significantly elevated PTK2 expression level in LSCC cells, revealing that the transfection was successful. Furthermore, we identified that miR‐135a‐5p mimics could downregulate PTK2 mRNA level, while this inhibitory effect was abolished by PTK2 overexpression (Figure 6C). Next, we investigated whether miR‐135a‐5p affects the PTK2/PI3K pathway to regulate LSCC progression. We found that miR‐135a‐5p decreased phosphorylation of PI3K (p‐PI3K) and PTK2 protein level (Figure 6D). Intriguingly, overexpression of PTK2 could reverse miR‐135a‐5p mimic‐mediated p‐PI3K downregulation (Figure 6D). Furthermore, MTT and colony formation results manifested that miR‐135a‐5p mimics obviously attenuated cell viability and proliferation; however, PTK2 overexpression enhanced cell viability and proliferation (Figure 6E,F). Consistently, miR‐135a‐5p mimics inhibited migration and invasion, which was hindered by overexpression of PTK2 (Figure 6G,H). Collectively, these findings indicated that miR‐135a‐5p overexpression antagonized the PTK2/PI3K pathway to inhibit LSCC malignant phenotypes.

## 4 DISCUSSION

LSCC is the most prevalent head and neck squamous cell carcinomas (HNSCC).^18^ Despite significant advances in LSCC therapy across the world, the 5‐year prognosis for LSCC patients remains unsatisfactory.^19^ Hence, it is critical to understand carcinogenesis mechanisms in order to create novel targeted therapeutics for LSCC. In this study, we found that AC004943.2 upregulation promoted LSCC progression. Mechanistically, AC004943.2 facilitated LSCC development by activating the PTK2/PI3K pathway through sponging miR‐135a‐5p.

Numerous studies have reported that lncRNAs play essential roles in LSCC progression.^20^, ^21^ It has been widely documented that lncRNAs acted as miRNAs sponges and competed with mRNAs,^22^ and the lncRNA‐miRNA‐mRNA network was essential for LSCC progression. For instance, the lncRNA FGD5‐AS1/miR‐497‐5p axis modulated SEPT2 to promote LSCC progression and enhanced cisplatin resistance.^20^ Li et al. reported that lncRNA SNHG20 accelerated the progression of LSCC by sponging miR‐140.^23^ A previous study demonstrated that lncRNA AC004943.2 was overexpressed in LSCC.^9^ Nonetheless, the exact function and the mechanism of lncRNA AC004943.2 in LSCC remain unclear. Our study revealed that AC004943.2 was highly expressed in LSCC cells, which was consistent with previous research.^9^ In addition, AC004943.2 silencing markedly suppressed LSCC cell viability, proliferation, and migration and invasion. Our results confirmed that AC004943.2 upregulation promoted the progression of LSCC.

Many studies have shown that miRNAs contribute to the development of LSCC.^24^, ^25^, ^26^ Erkul et al. found that miR‐21 acted as a crucial factor in diagnosis and serve as a possible biomarker in LSCC.^27^ The function of miR‐135a‐5p in cancers has been widely studied.^28^, ^29^ However, further research is needed to determine the exact function and the mechanism of miR‐135a in LSCC. Our findings discovered that miR‐135a‐5p was lowly expressed in LSCC, and its overexpression could suppress LSCC cell malignant behaviors. Furthermore, we found that AC004943.2 reduced miR‐135a‐5p expression in LSCC cells by sponging miR‐135a‐5p. As expected, miR‐135a‐5p inhibition reversed the inhibitory effects of AC004943.2 silencing on LSCC cell malignant behaviors. Collectively, our research indicated that AC004943.2 upregulation accelerated LSCC development by sponging miR‐135a‐5p.

PTK2, a non‐receptor tyrosine kinase, plays vital roles in diverse cellular processes, such as growth factors signaling, cell cycle, cell survival, angiogenesis, and immunosuppressive tumor microenvironments.^30^ PTK2 is highly expressed in a variety of cancer types.^30^ Notably, previous investigations have demonstrated that PTK2 was involved in the progression of LSCC. For instance, AURKA revived dormant LSCC via activating the PTK2/PI3K/Akt pathway.^31^ ROCK1 facilitated LSCC tumorigenesis and progression by mediating the PTK2 signaling pathway.^32^ We also found that PTK2 was overexpressed in LSCC cells. MiRNAs are negative posttranscriptional regulators of target gene expression.^33^ We confirmed that PTK2 was the target gene of miR‐135a‐5p. Furthermore, we found that miR‐135a‐5p overexpression markedly reduced PTK2 expression in LSCC cells. Taken together, we firstly discovered that miR‐135a‐5p suppressed LSCC progression via attenuating the PTK2/PI3K pathway.

In summary, our findings demonstrated that AC004943.2 increased PTK2 via sponging miR‐135a‐5p and thus activated the PTK2/PI3K pathway to promote the tumorigenesis of LSCC. Our research is expected to provide valuable insights into elucidating the molecular mechanism of AC004943.2 in LSCC.

## AUTHOR CONTRIBUTIONS

**Xiaowen Zhu**: Conceptualization; methodology; validation; formal analysis; investigation; resources; data curation. **Wenming Dong**: Writing – original draft; visualization; supervision. **Meijia Zhang**: Writing – review & editing; project administration; funding acquisition.

## CONFLICT OF INTEREST STATEMENT

The authors declare that they have no conflict of interest.

## ETHICS STATEMENT

Ethics statement not applicable to this article as this article covers only cell experiments, and all cell lines are commercially available.

## Data Availability

Data sharing is not applicable to this article as no datasets were generated or analyzed during the current study.
